# Endoscopic diverticulectomy and myotomy for epiphrenic diverticulum

**DOI:** 10.1016/j.vgie.2024.12.003

**Published:** 2024-12-15

**Authors:** Fatih Aslan, Serhat Ozer, Burcu Saka, Akın Akbulut, Furkan Kartal

**Affiliations:** 1 Department of Gastroenterology and Advanced Endoscopy, Koc University Hospital, Istanbul, Turkey; 2 Department of Pathology, Koc University Hospital, Istanbul, Turkey; 3 Department of Anesthesiology and Reanimation, Koc University Hospital, Istanbul, Turkey; 4 Department of Radiology, Koc University Hospital, Istanbul, Turkey

Epiphrenic diverticula (ED), also known as pulsion diverticula, are a rare abnormality located in the distal 10 cm of the esophagus or 4 to 8 cm above the gastric cardia and are mostly accompanied by an esophageal motility disorder. The mechanism involves herniation of mucosa, muscularis mucosa, and submucosa through weakened muscularis propria mostly due to increased distal esophageal pressure secondary to achalasia, hypertensive lower sphincter, or tight fundoplication.^1^^,^^2^ Novel minimally invasive approaches, as a part of third-space endoscopy, are now evolving, while thoracotomy remains the primary treatment.^3^^,^^4^ We present herein a case of symptomatic ED treated endoscopically.

## Case

**Figure 1:**
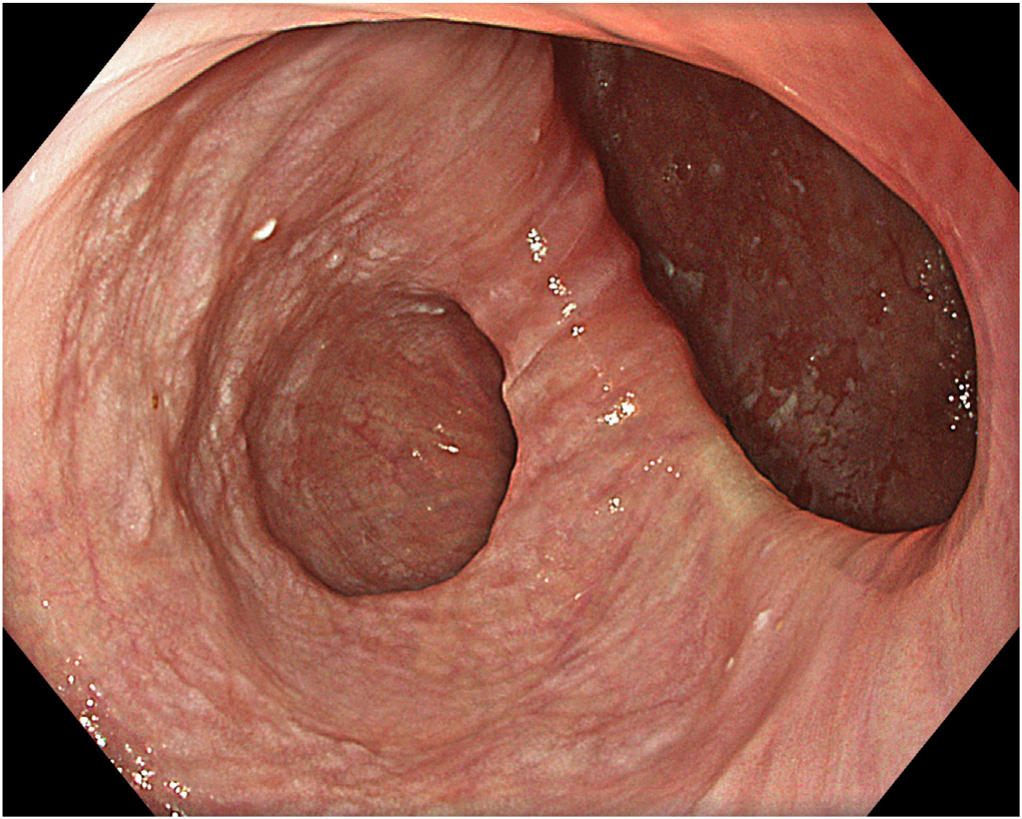
Endoscopic view of the esophageal lumen and the epiphrenic diverticulum.

**Figure 2:**
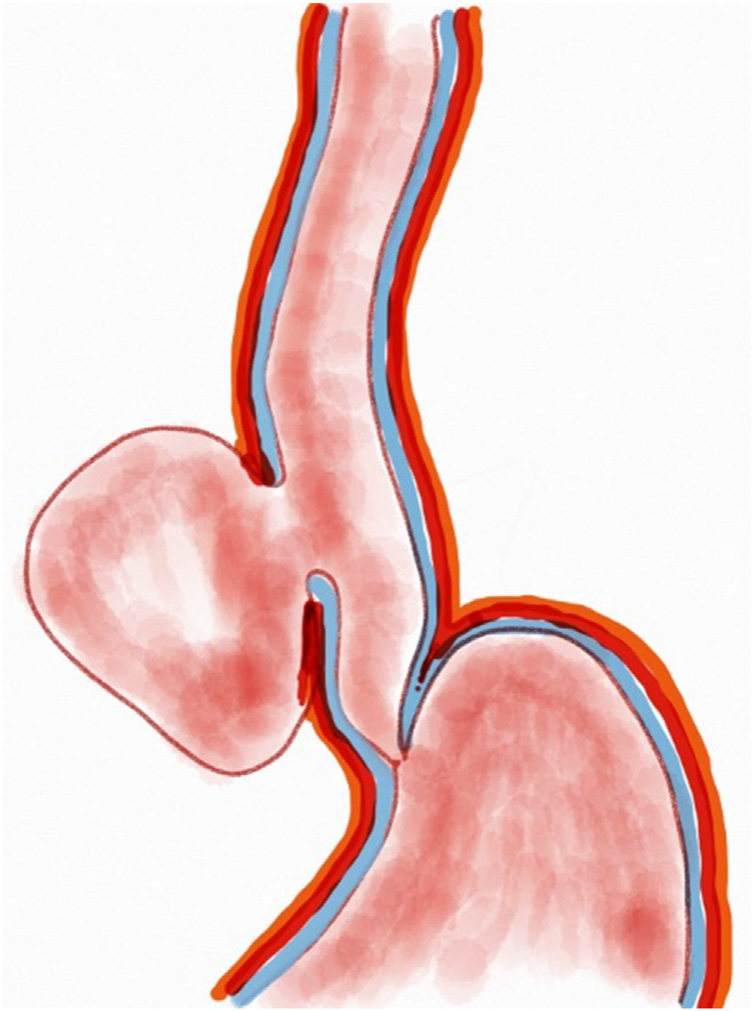
Schematic view of the epiphrenic diverticulum.

**Figure 3:**
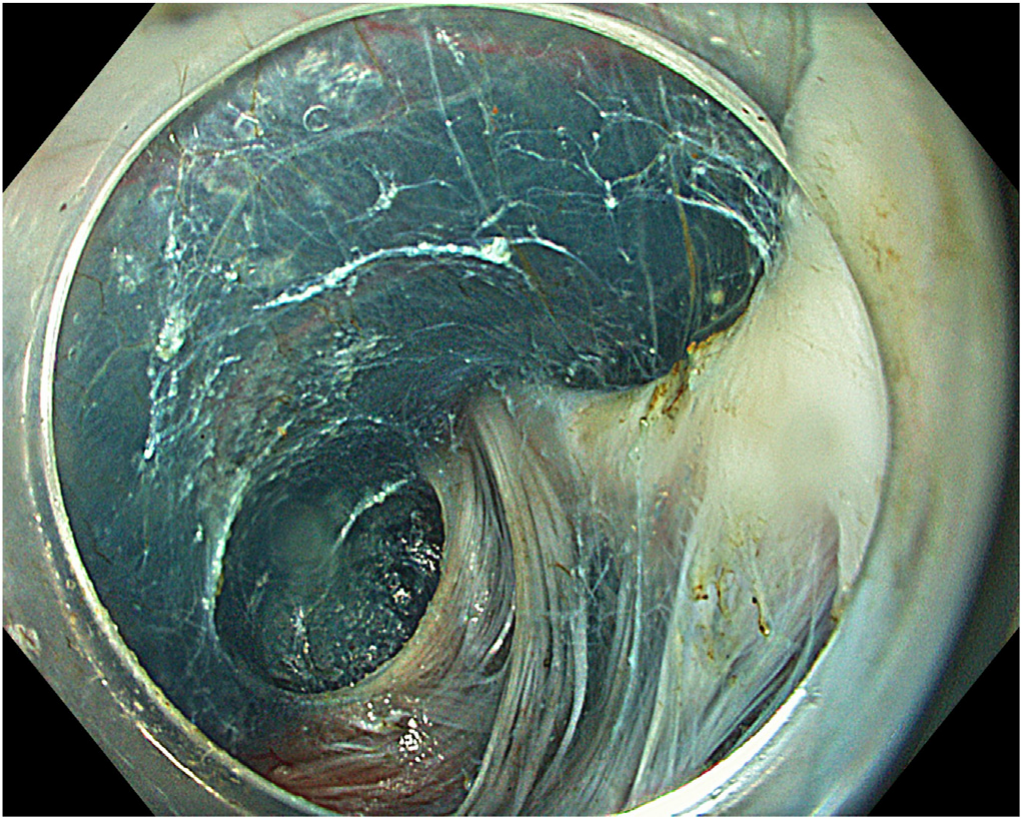
Endoscopic view of the posterior part of the diverticulum opening from within the tunnel.

**Figure 4:**
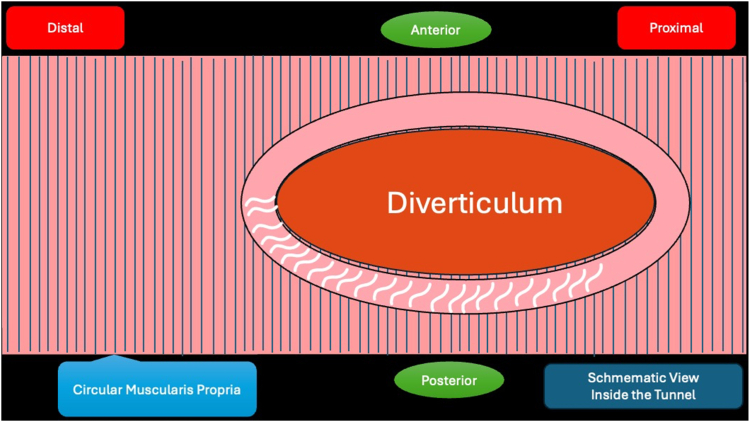
Schematic view of the posterior part of the diverticulum opening from within the tunnel.

**Figure 5:**
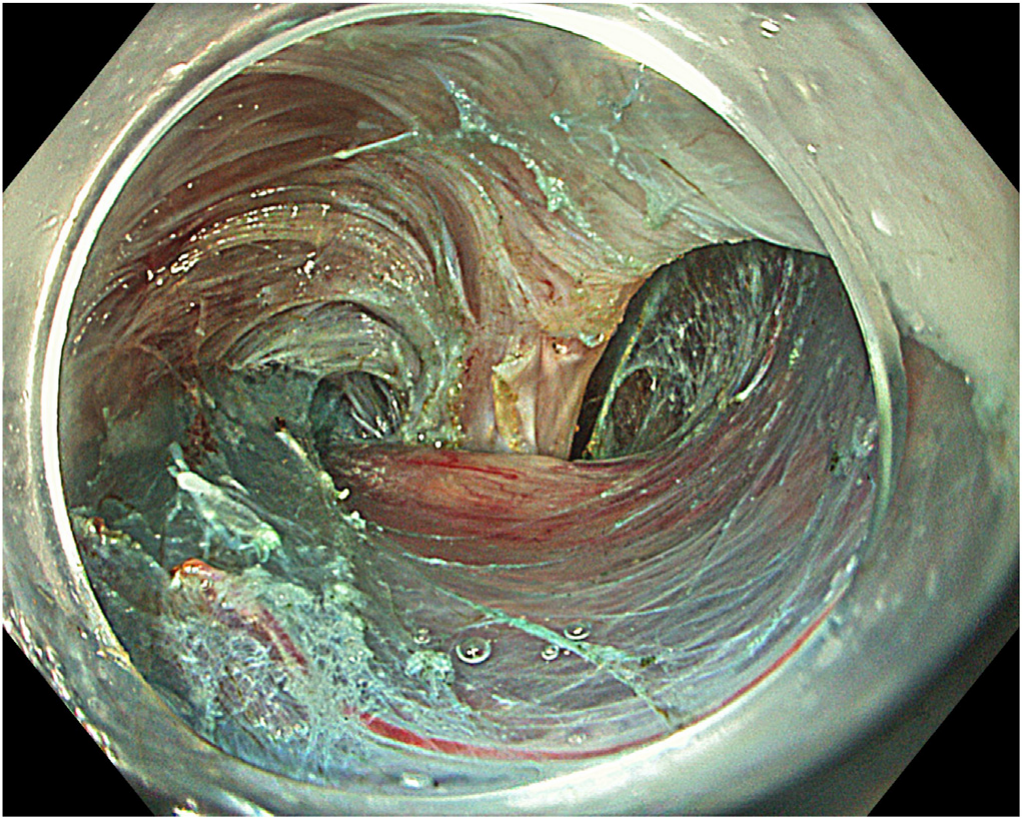
Endoscopic view of the anterior part of the diverticulum opening from within the tunnel.

**Figure 6:**
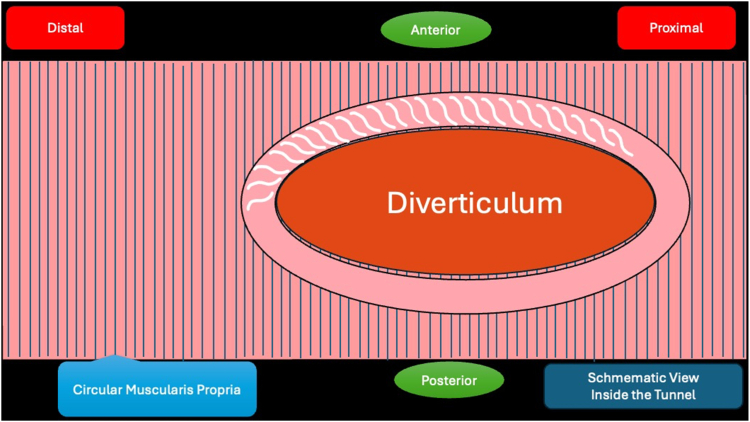
Schematic view of the anterior part of the diverticulum opening from within the tunnel.

**Figure 7:**
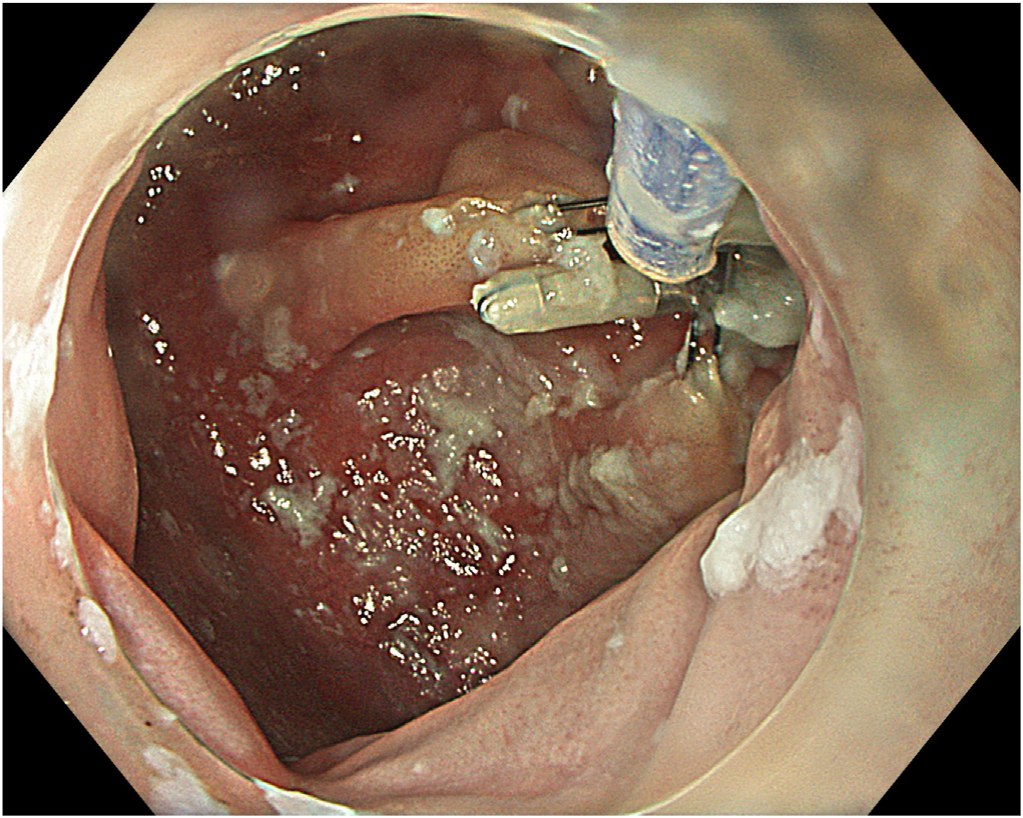
Endoscopic view of the diverticulum being pulled into traction with the help of a snare and clip.

**Figure 8:**
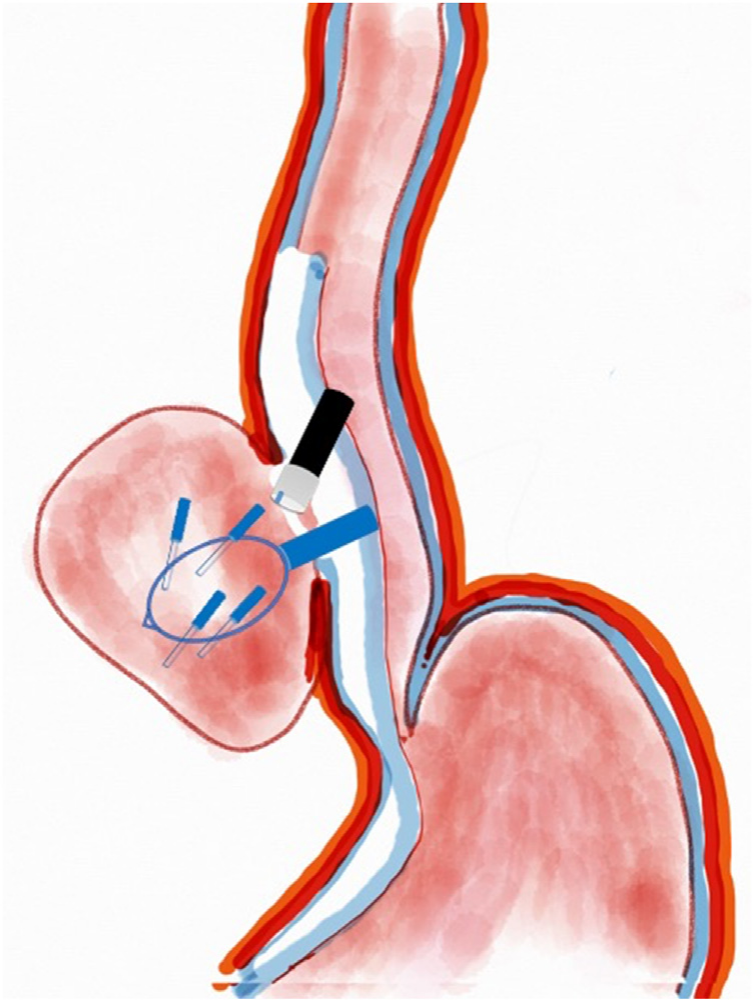
Schematic view of the diverticulum being pulled into traction with the help of a snare and clip.

**Figure 9:**
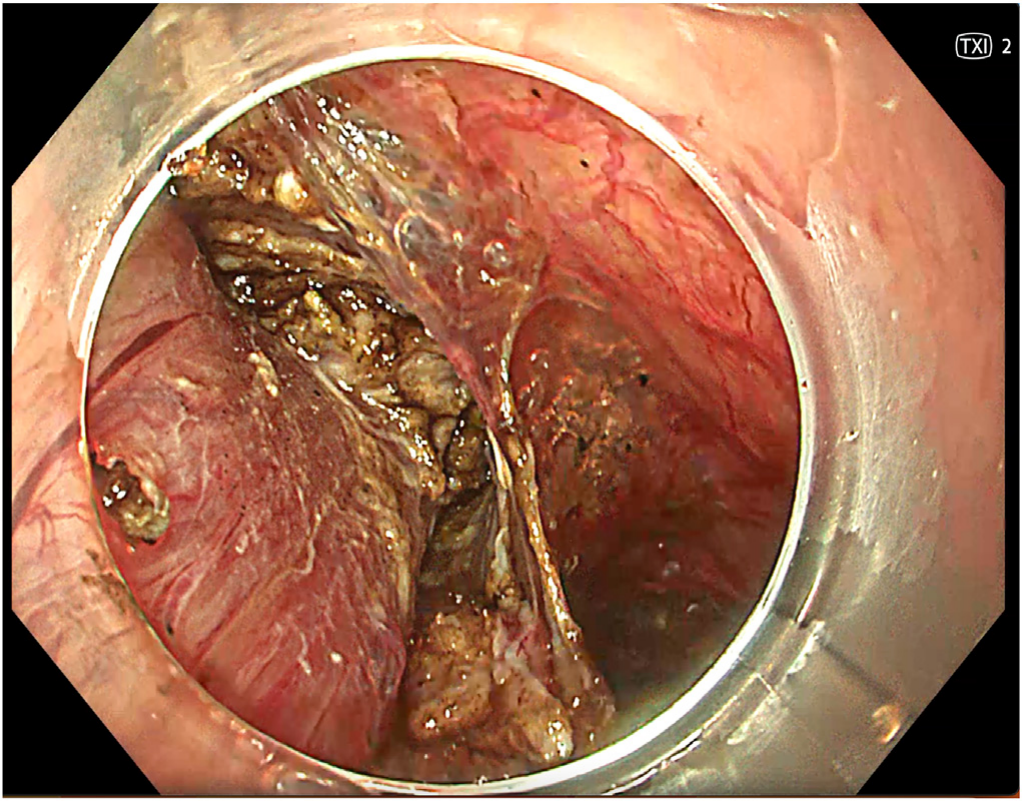
Endoscopic view of the diverticulum septum and distal myotomy application.

**Figure 10:**
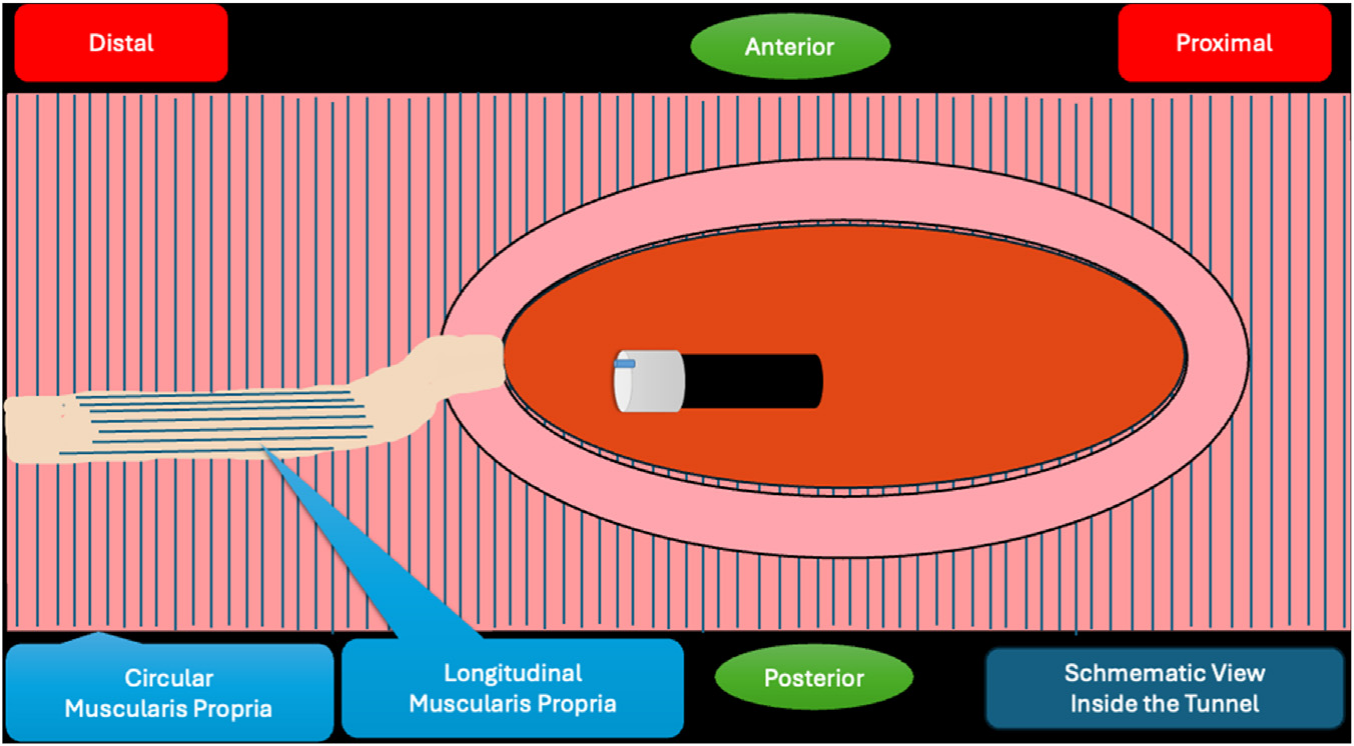
Schematic view of the diverticulum septum and distal myotomy application.

A 53-year-old female patient presented with dysphagia, chest pain, cough, and vomiting. Barium swallow test and chest CT revealed a lumen-narrowing well-defined epiphrenic diverticulum 9 cm in size plus an achalasia-like appearance in the lower esophageal sphincter. Endoscopy confirmed the diverticulum (Figs. 1 and 2). High-resolution manometry could not be performed because of the failure of catheter insertion through the lower sphincter; however, pan-esophageal pressurization was noted. As given consent was taken, peroral endoscopic myotomy (POEM) along with endoscopic diverticulum resection was scheduled. Submucosal injection with indigo carmine and saline solution was performed 5 cm above the diverticulum in a 5 o’clock position using a sclerotherapy needle (NeedleMaster; Olympus, Tokyo, Japan). Submucosal space was entered after linear mucosal incision with a triangle-tip knife (Olympus) under certain cautery settings (pulse-cut slow, 40-W, effect 2; spray-coag 40-W, effect 2) (Olympus ESG300). A posterior submucosal tunnel up to distal to gastroesophageal junction, including posterior edge of diverticulum, was opened followed by a second tunnel created between 1 cm distal to mucosal incision and anterior entrance of the diverticulum (Figure 3, Figure 4, Figure 5 to 6). The diverticular base was reached as anterior and posterior entrances were merged within the tunnel, and the remaining uncompleted part was dissected as the diverticulum was inverted into the lumen using a snare. A 30-mm standard polypectomy snare (Steris Lariat Snare 30 mm, Dublin, Ireland) was taken out of the endoscope to the luminal aspect of the diverticulum and attached using hemostatic clips (EZ clip; Olympus). The diverticulum was inverted for fast and safe dissection as the snare was closed (Figs. 7 and 8). As the diverticulum freed, hemostatic clips were detached, and grasping forceps (Ovesco, Tübingen, Germany) sent through the endoscope were attached instead, followed by snare retrieval brought out of the endoscope for a tighter and safer traction (Video 1, available online at www.videogie.org) (Figs. 9 and 10). Afterward, septotomy was performed, followed by posterior selective circular and full-thickness myotomy for GE junction and GE junction to tunnel ending, respectively.

**Figure 11:**
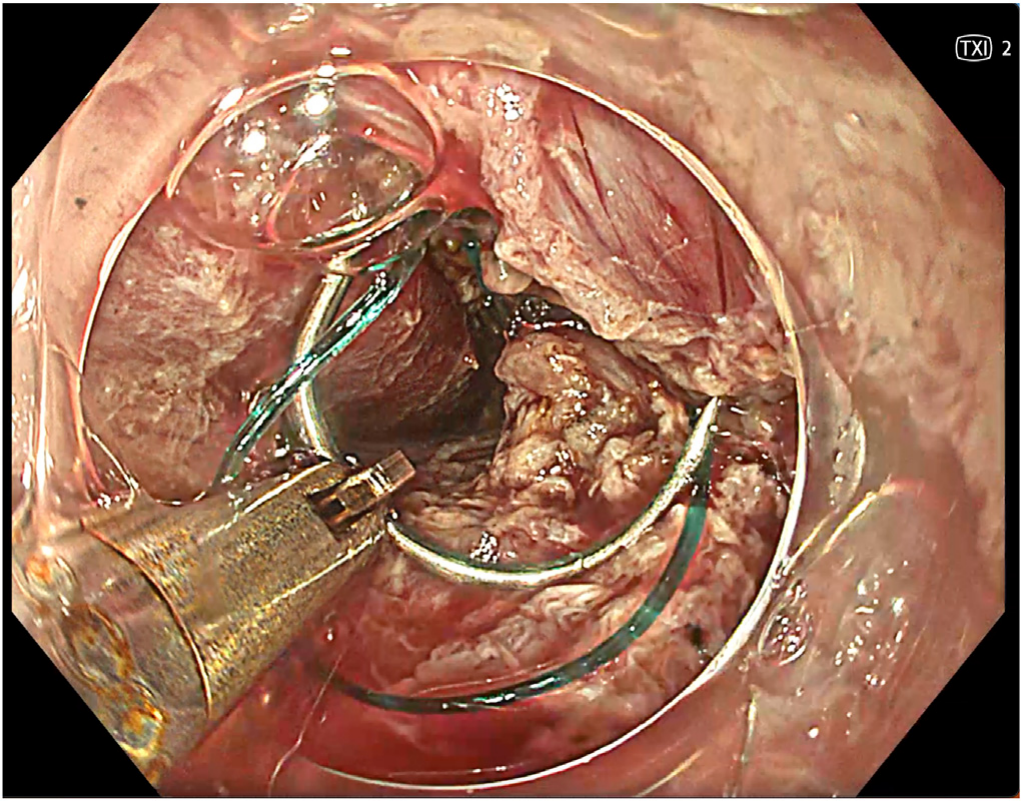
Endoscopic view of the application of barbed sutures to the diverticulum opening.

**Figure 12:**
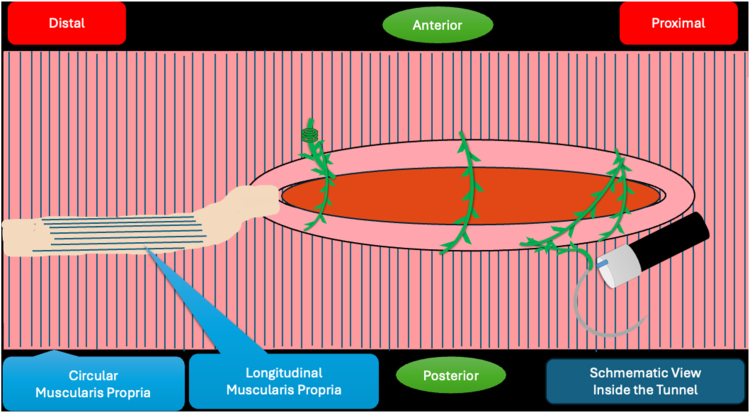
Schematic view of the application of barbed sutures to the diverticulum opening.

**Figure 13:**
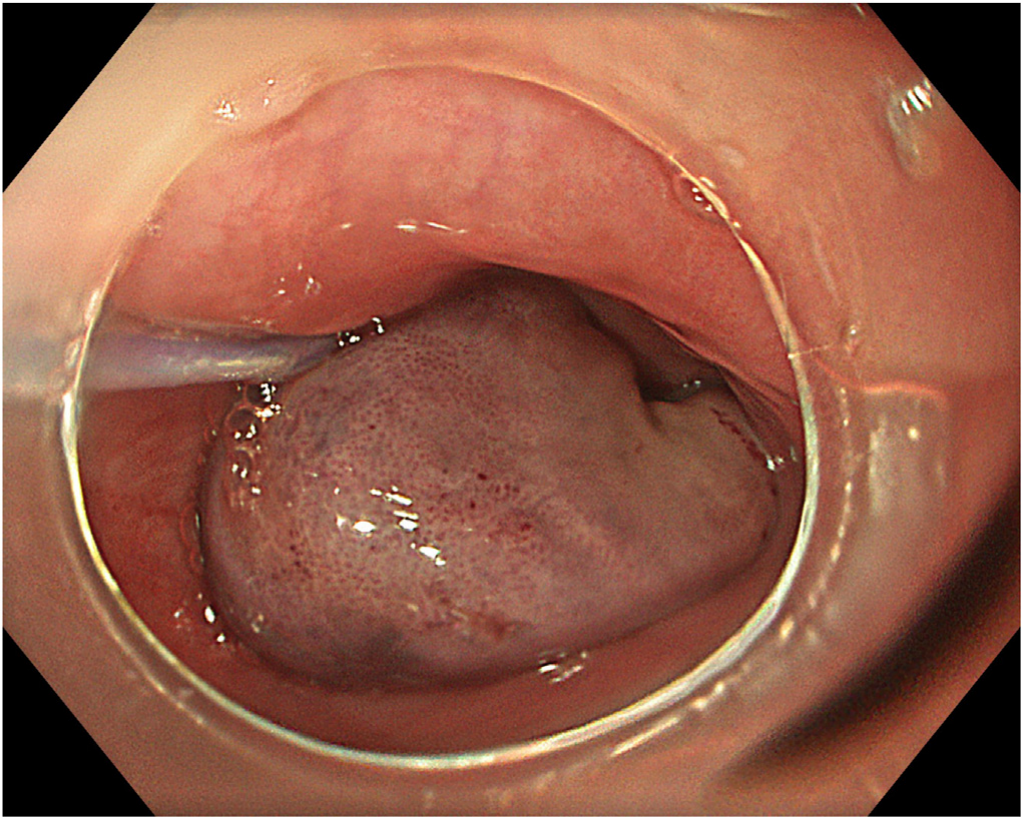
Removal of the diverticulum, which has been inverted into the lumen with the help of a snare.

**Figure 14:**
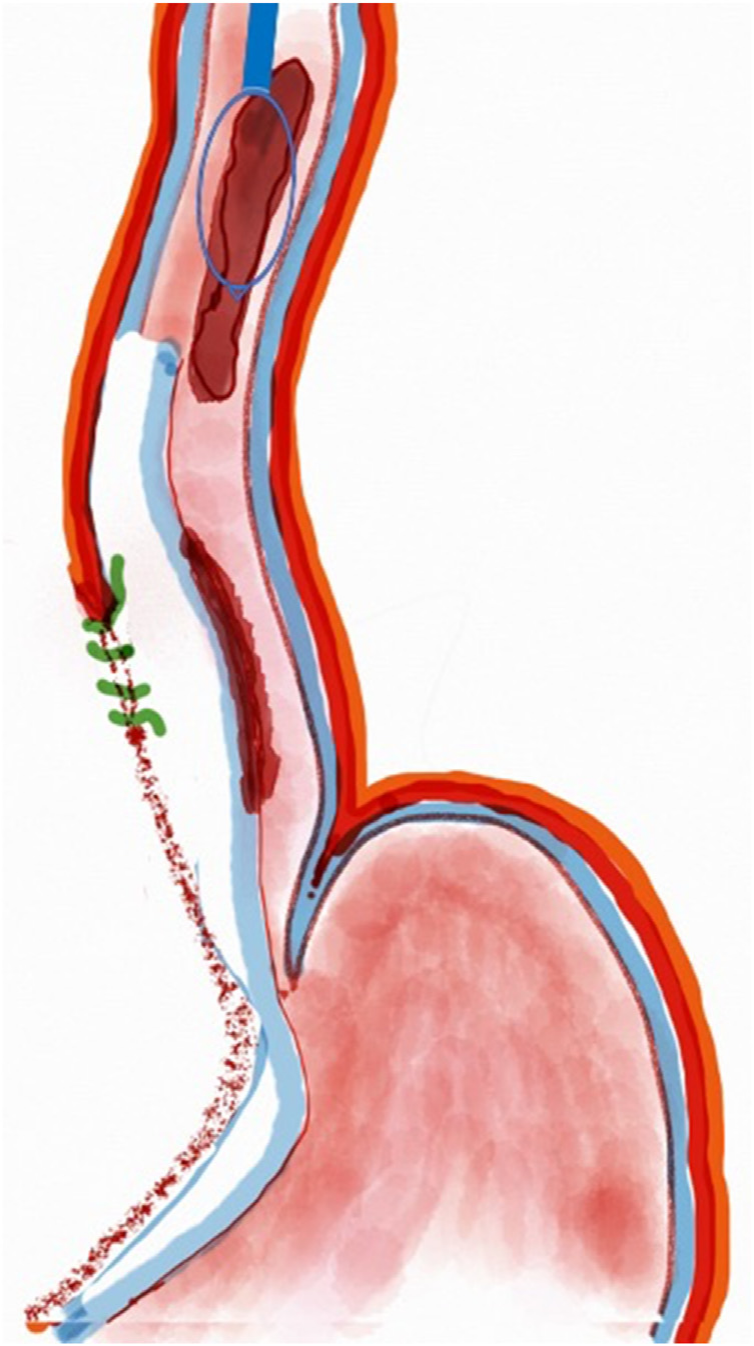
Schematic view of removal of the diverticulum, which has been inverted into the lumen with the help of a snare.

**Figure 15:**
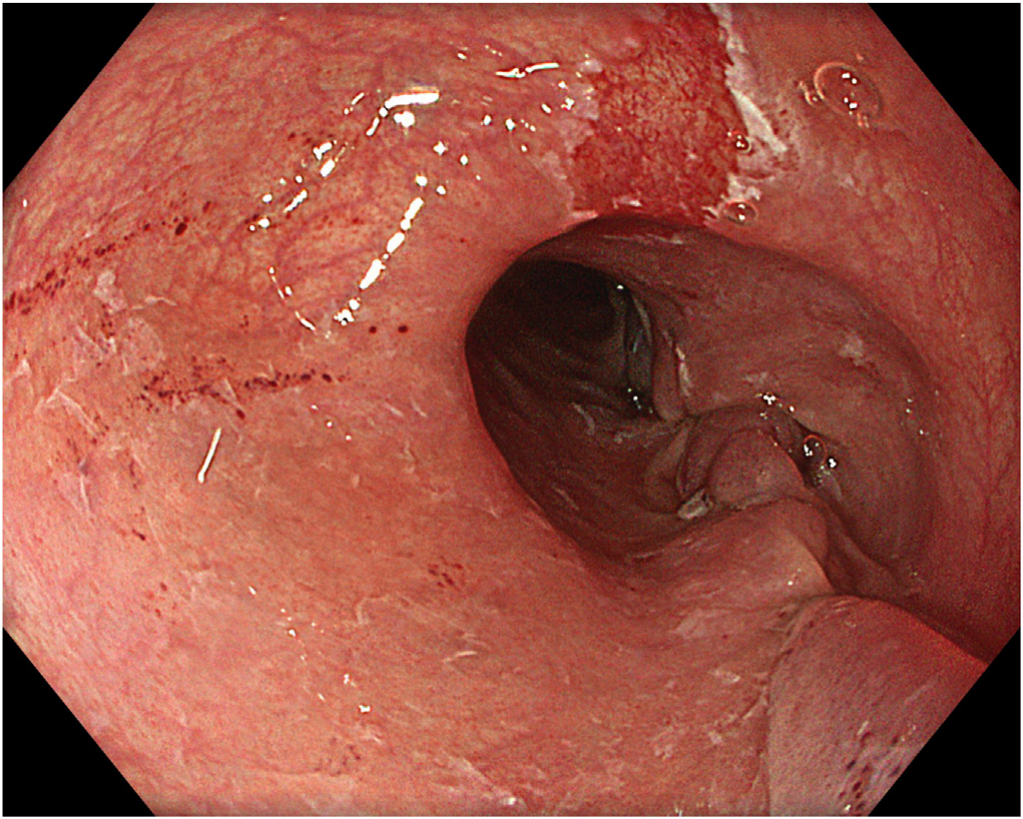
Endoscopic view of the area where diverticulectomy was performed and the tunnel entry with the application of a barbed suture.

**Figure 16:**
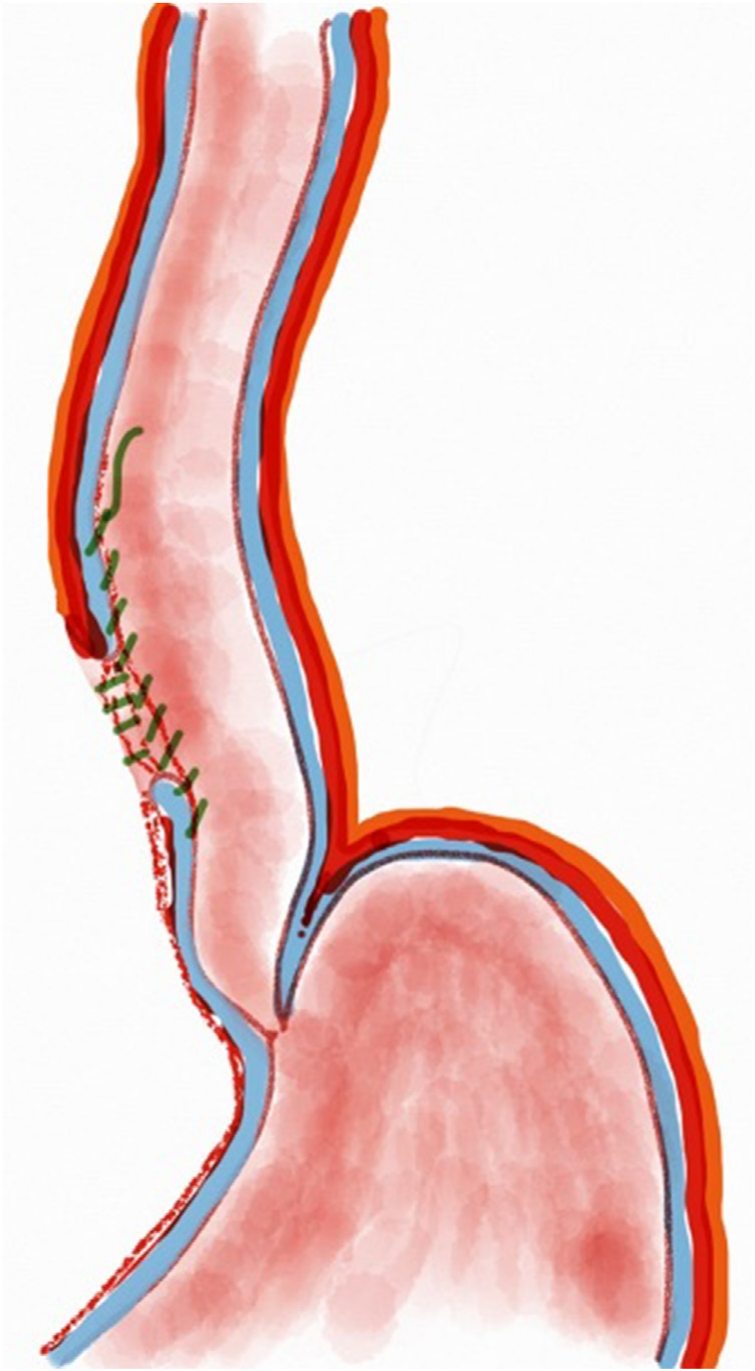
Schematic view of the area where diverticulectomy was performed and the tunnel entry with the application of a barbed suture.

A barbed suture (V-Loc 180, 2-0, CV-23; Medtronic Ltd, Dublin, Ireland), 15 cm in length at baseline, was shortened as the distal part cut and knotted to prevent confusion in narrow submucosal space and taken to the tunnel in a distal attachment (Olympus) using the Needle Holder (Sutuart, FG 260U; Olympus) through the working channel. Anterior and posterior muscular edges of the diverticulum were first stitched with 3 consecutive running sutures and locked as the last suture crossing the previous one before diverticular resection to lower the risk of contamination of mediastinal space (Figs. 11 and 12). Excess suture material was cut and removed (Loop Cutter; Olympus). The next step was to resect diverticular tissue under traction with standard snare polypectomy (Figs. 13 and 14). The resultant mucosal defect and tunnel entrance were closed using the previous barbed suture with 14 insertions in a continuous fashion and locked as the last insertion crossing the previous one. Again, excess suture material was cut and removed, and esophageal integrity and reconstruction were provided (Figs. 15 and 16).

**Figure 17:**
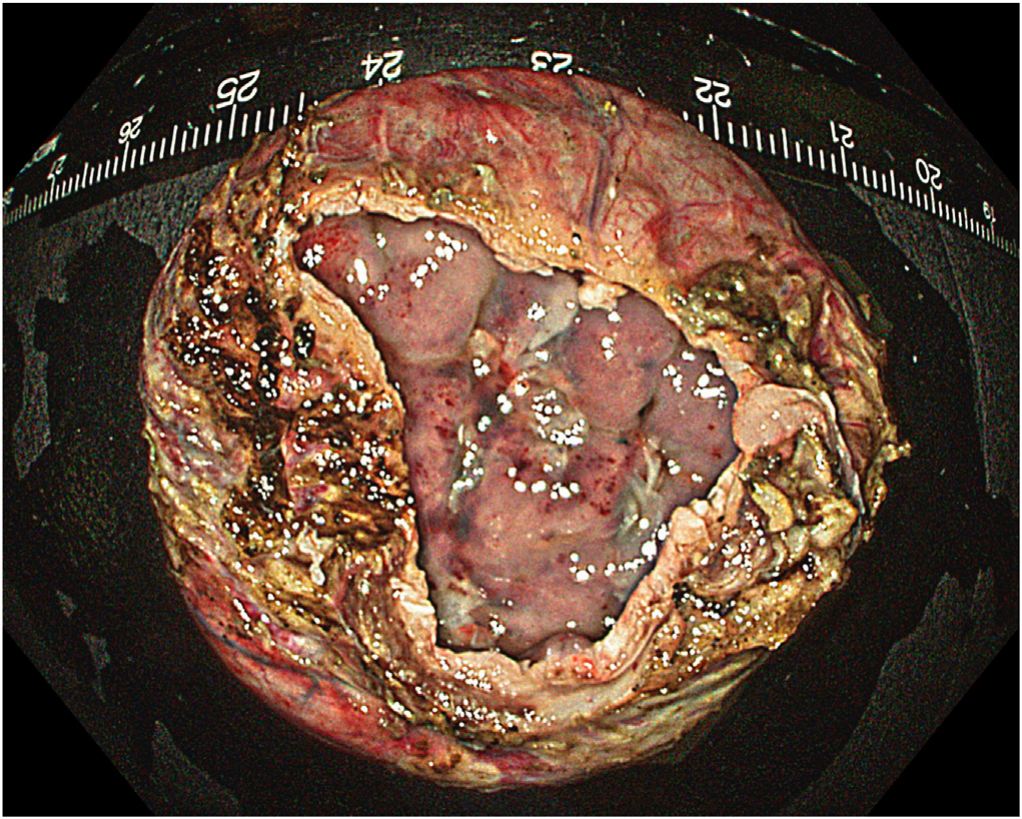
Macroscopic view of the endoscopic diverticulectomy specimen (normal position).

**Figure 18:**
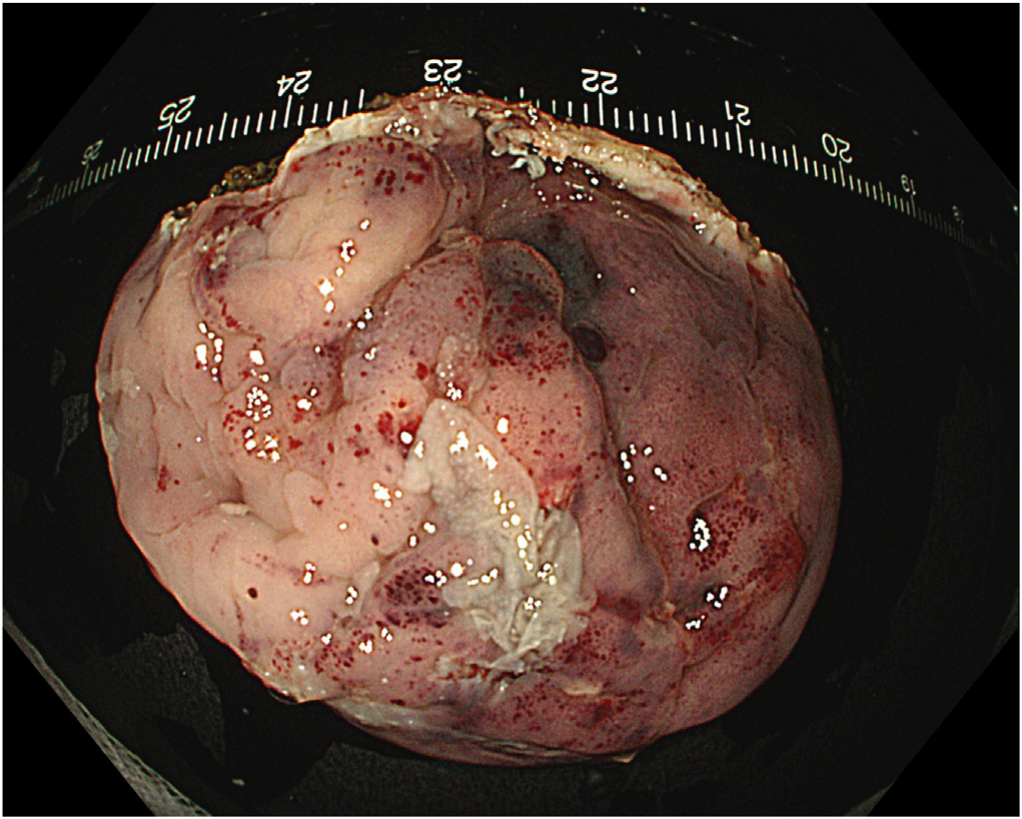
Macroscopic view of the endoscopic diverticulectomy specimen (inverted position).

**Figure 19:**
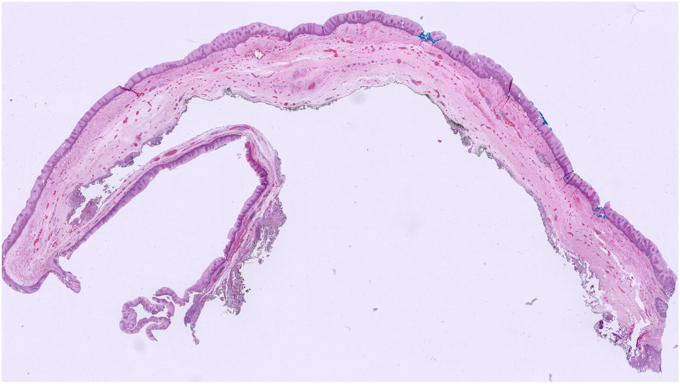
Microscopic view of the endoscopic diverticulectomy specimen (H&E, orig. mag ×2).

**Figure 20:**
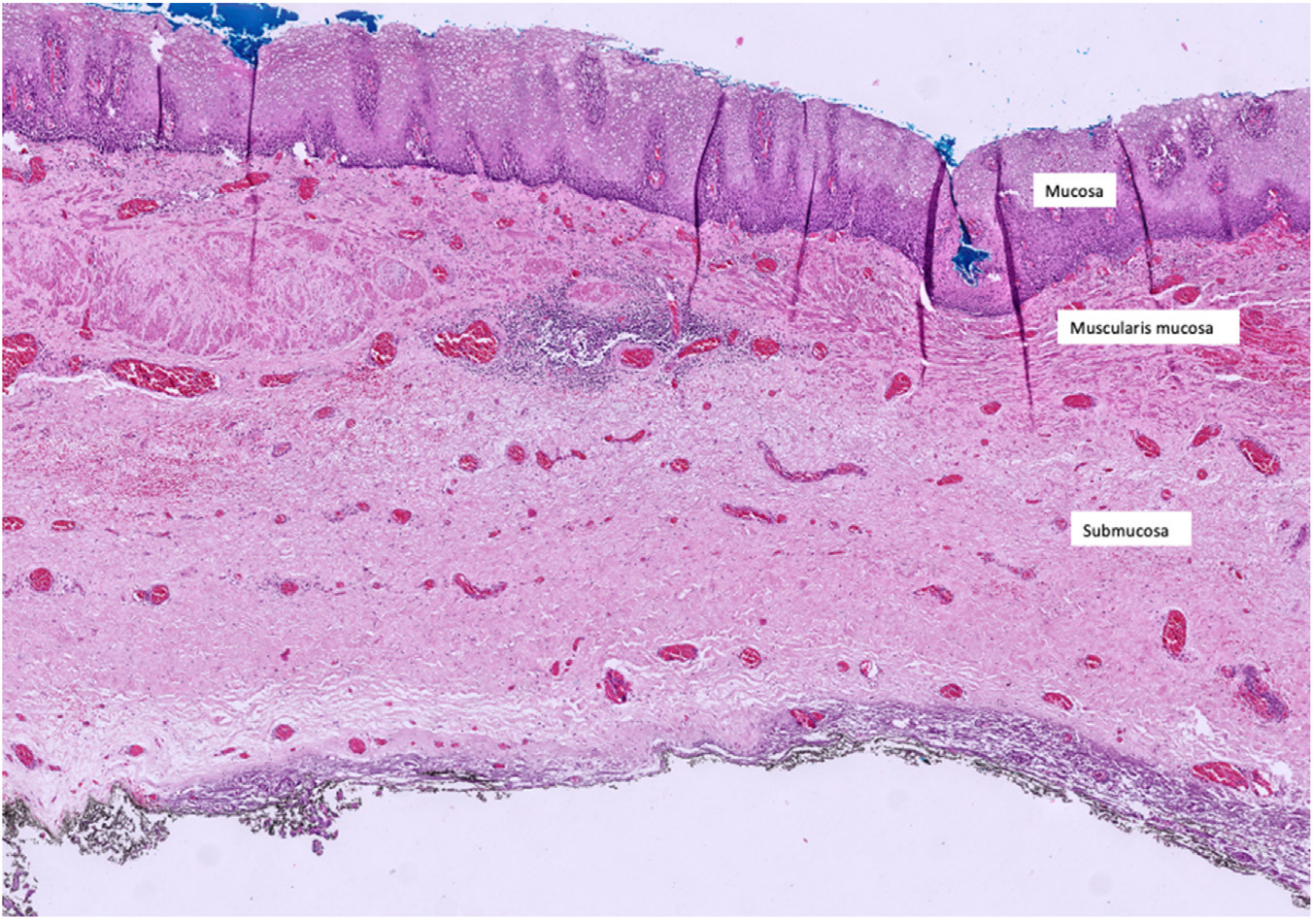
Microscopic view of the endoscopic diverticulectomy specimen (H&E, orig. mag ×40).

No adverse event was noted during the procedure. To prevent tension pneumothorax due to free carbon dioxide (CO_2_) in abdominal space after myotomy, a puncture with a 14-gauge peripheral venous catheter was applied 2 fingers above the umbilicus when clinically significant distension occurred, and peak pressure increased, and was kept in place to provide stable pulmonary ventilation. Piperacillin-tazobactam was started given mediastinal contamination and administered during in-hospital stay. The chest radiograph taken on day 2 showed minimal atelectasis and pleural effusion, which were self-limited. Patients were on nothing-by-mouth for 2 days, and oral intake was resumed gradually by day 3. Barium swallow test showed no diverticulum, and once the serum C-reactive protein (CRP) level fell below 10 mg/dL, the patient was discharged by day 7 (Video 1, available online at www.videogie.org). Pathology resulted as diverticular tissue consisting of mucosa, muscularis mucosa, and submucosa, both macroscopically and microscopically (Figure 17, Figure 18, Figure 19 to 20).

## Discussion

Thoracotomy with robotic or laparoscopic approaches is widely used in ED. However, morbidity and recurrence rates are reported as 5% to 37% and above 20%, respectively.^5^ POEM has been a well-established treatment of choice in achalasia and jackhammer esophagus. Dysmotility is well known to play a role in ED. Thus, POEM has been tried with favorable results.^6^ However, in cases in which POEM alone is applied, the diverticulum itself remains and acts as a reservoir although intradiverticular food retention resolves. In our case, the diverticulum was first released and inverted into the lumen; then POEM was applied to decrease pressure. Then, the muscular layer proximal and distal to the diverticulum was merged to prevent recurrence and to lower contamination. Afterward, excessive diverticular tissue was resected and removed, and a closure with complete mucosal integrity was provided using the barbed suture. Endoscopic reconstruction was proven to be successful in radiological studies.

During POEM and diverticulectomy, as myotomy is performed, CO_2_ can easily diffuse mediastinum and intra-abdominal space. Hemodynamic stability is well provided with drainage of free CO_2_ during the procedure through the puncture.^7^ In this case, with a similar approach, decompression was provided, and no adverse event was noted. Another possible adverse event is infection. A broad-spectrum antibiotic was started preoperatively given forecasted mediastinal contamination and was continued till discharge. However, although no postoperative fever was seen and leakage was noted in radiological workup, CRP fell below 10 mg/dL on day 6, which delayed the discharge to day 7.

Treatment of ED may change in favor of endoscopy as the use of sutures, which enables safe closure in advanced endoscopic procedures, increases. Use of POEM and endoscopic diverticulectomy, combined with advanced traction and suturing methods, may provide a novel treatment in appropriate cases.

## Disclosure

The authors have no financial relationship(s) to disclose.

## Supplementary data

Video 1 Endoscopic diverticulectomy and myotomy for epiphrenic diverticulum (7.59 seconds).
